# Large Scale Synthesis and Light Emitting Fibers of Tailor-Made Graphene Quantum Dots

**DOI:** 10.1038/srep14163

**Published:** 2015-09-18

**Authors:** Hun Park, Sung Hyun Noh, Ji Hye Lee, Won Jun Lee, Jae Yun Jaung, Seung Geol Lee, Tae Hee Han

**Affiliations:** 1 Department of Organic and Nano Engineering, Hanyang University, Seoul 04763, Republic of Korea; 2 Department of Organic Material Science and Engineering, Pusan National University, Busan 46241, Republic of Korea; 3 Department of Chemistry, Imperial College London, London, SW7 2AZ, United Kingdom

## Abstract

Graphene oxide (GO), which is an oxidized form of graphene, has a mixed structure consisting of graphitic crystallites of sp^2^ hybridized carbon and amorphous regions. In this work, we present a straightforward route for preparing graphene-based quantum dots (GQDs) by extraction of the crystallites from the amorphous matrix of the GO sheets. GQDs with controlled functionality are readily prepared by varying the reaction temperature, which results in precise tunability of their optical properties. Here, it was concluded that the tunable optical properties of GQDs are a result of the different fraction of chemical functionalities present. The synthesis approach presented in this paper provides an efficient strategy for achieving large-scale production and long-time optical stability of the GQDs, and the hybrid assembly of GQD and polymer has potential applications as photoluminescent fibers or films.

Graphene oxide (GO) is separated from the bulk graphite powders via a chemical oxidation process^1^,^2^, and is therefore decorated with a variety of oxygen-containing functional groups such as hydroxyl, epoxide, and carboxylic groups^3^. Unlike the highly crystalline graphene, GO is viewed as a unique 2D random block copolymer with both crystalline and amorphous defect regions, owing to the presence of sp^3^ carbon and oxygen functional groups^4^,^5^. However, the defect sites on GO have blocked practical application of GO materials due to insufficient electrical conductivity^4^,^6^. In this regard, many strategies such as thermal annealing^7^, vapor-mediated reduction^8^, and hetero-atomic doping^9^,^10^,^11^ have been studied to overcome those drawbacks without losing superior chemical reactivity of GO. Different from the conventional defect restoration approaches^12^,^13^,^14^,^15^, our steam activation method has been appreciated for its selective decomposition of the defective areas with less energy, leading to nanoporous GO^6^. In this strategy, controlling the defects in GO provides a useful tool for controlling its morphology, thereby offering an efficient route for the preparation of functional carbon nanomaterials. Furthermore, complete elimination of defects is possible, which would leave behind graphitic domains that are optically active functional nanomaterials.

Graphene-based quantum dots (GQDs) have been extensively investigated owing to the various advantages they possess, including non-toxicity, easy synthesis procedures, and controllable chemical functionality^16^,^17^,^18^,^19^,^20^,^21^. So far, several approaches including molecular assembly of carbon ring structures^22^,^23^, chemical exfoliation of graphite nanofibers^17^,^24^, modification of graphite nanoparticles^25^, and a hydrothermal route resulting in the fracture of GO sheets into ultra-small pieces^26^,^27^,^28^,^29^, have been studied to synthesize GQDs. However, challenges such as low synthesis yields resulting from the formation of highly aggregated graphene monoliths, still exist and need to be overcome^18^,^26^. In addition, poor optical properties such as low quantum yield (QY), poor control of the emission wavelength and optical instability, limit direct applications^30^,^31^.

Herein, we introduce a one-step synthesis procedure for producing GQDs with high QY and long-term optical stability via the extraction of GQDs from GO. GQDs were prepared with high production yields by using small amounts of environmentally benign chemicals, such as H_2_O_2_ and NH_3_. Additionally, when employed together, these chemicals effectively extracted GQDs from GO, and simultaneously decorated GQDs with controllable functionalities. The controlled introduction of functional groups was achieved by changing the reaction temperature, which resulted in the ability to precisely tune their optical properties. Our strategy, namely the selective elimination of defects in GO sheets, offers a novel pathway to produce GQDs from the bulk graphite powders in large quantities with high QY and also enables the production of novel GQDs-based polymeric composites, such as photoluminescent fibers and films.

## Results and Discussion

**Figure 1:**
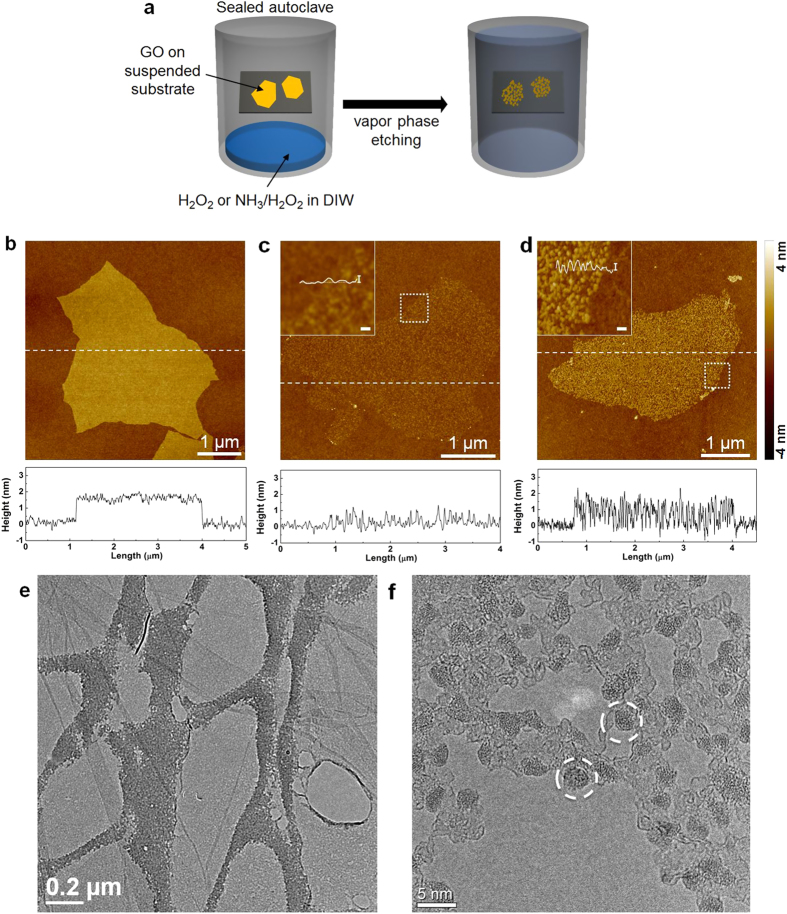
(**a**) Schematic diagram showing the vapor-phase etching process on GO. First, GO monolayers were deposited on substrates by Langmuir-Blodgett technique, following which the substrates were suspended in a sealed vessel prefilled with the etching solution mixture. The vessel was then heated at 150 °C to allow the vapor-phase reaction to occur. AFM images of (**b**) pristine GO and GO sheets etched with a H_2_O_2_/DIW solution mixture for (**c**) 30 min. The corresponding height profiles are measured along the white lines. (**d**) AFM image of a GO sheet etched with a NH_3_/H_2_O_2_/DIW solution mixture for 30 min. All the scale bars in the insets represent 1 nm for height (I) and 100 nm for length (−). (**e**,**f**) TEM images of steam-activated GO sheets suspended on a lacey carbon grid at (**e**) low magnification and (**f**) high magnification.

A schematic illustration of vapor-phase etching on single-layer GO sheets, which serves as a tool for directly observing the formation of GQDs, is shown in Fig. 1a. Firstly, single GO layers were coated on SiO_2_/Si substrates using the Langmuir-Blodgett technique^32^. Subsequently, each substrate was suspended in a Teflon vessel prefilled with an aqueous solution, which acted as the vapor source. The vessel was then sealed in a stainless steel autoclave and heated at 150 °C. Figure 1b shows a topographic image of the deposited GO sheets. The corresponding height profile indicates that the thickness of the GO sheets is about 1.6 nm. After the GO sheets were exposed to H_2_O_2_ vapor, which is widely known as peroxide etchant, for 25 min, pores were evenly generated over the GO sheets, as shown in Figure S1a. The corresponding height profile also confirms the generation of pores on the GO sheets. However, the GO sheets almost entirely disappeared with a few additional minutes (5 min) of vapor treatment (Fig. 1c) and its thickness was significantly reduced to less than 1 nm, indicating the chemical decomposition of GO. Compared to our previous report on steam-activated GO^6^, the use of peroxide etchant resulted in a fast etching reaction of the GO sheets. The vigorous reaction rate of H_2_O_2_ can be retarded by addition of a small amount of NH_3_, since NH_3_ provides a basic pH condition that induces the chemical decomposition of H_2_O_2_ into H_2_O and O_2_^33^,^34^. Figure 1d shows an AFM image of GO acquired after vapor etching with a solution mixture containing NH_3_, H_2_O_2_, and deionized water (DIW) for 30 min at 150 °C. Interestingly, in contrast to the H_2_O_2_-treated GO, the GO etched with above solution mixture was not greatly decomposed and still retained the porous structure. The inset in Fig. 1d indicates that the porous GO sheet consisted of many isolated dots of an average thickness of 1.6 nm, which were distributed homogeneously throughout the basal plane of the sheet. The observations in Fig. 1 indicate that the NH_3_/H_2_O_2_/DIW solution mixture is an efficient etchant for generating finite-sized dots in GO.

To further investigate the crystalline properties of the remaining dots, GO sheets loaded on a carbon-coated transmission electron microscopy (TEM) grid were treated with steam for 20 h at 200 °C and their crystalline morphologies were characterized using TEM. Figure 1e shows an image of the GO sheets suspended on a lacey carbon grid after steam-activation. Perforated GO sheets with nanopores were clearly observed even at low magnification (Fig. 1e). At higher magnifications (Fig. 1f), highly crystalline particles (which would be GQDs), as indicated with white dot circles, were clearly noticed as the pores grew preferentially along the amorphous matrix. Figure S1b shows that the electron diffraction pattern of steam-etched GO network containing GQDs (left) is similar to that of the GO crystalline structure (right). Evolution of pores indicates that the amorphous defective areas of sp^3^ carbon or oxygenated sites disappeared during the etching process. Indeed, this implies that GO sheets contain crystalline GQDs within an amorphous matrix.

**Figure 2:**
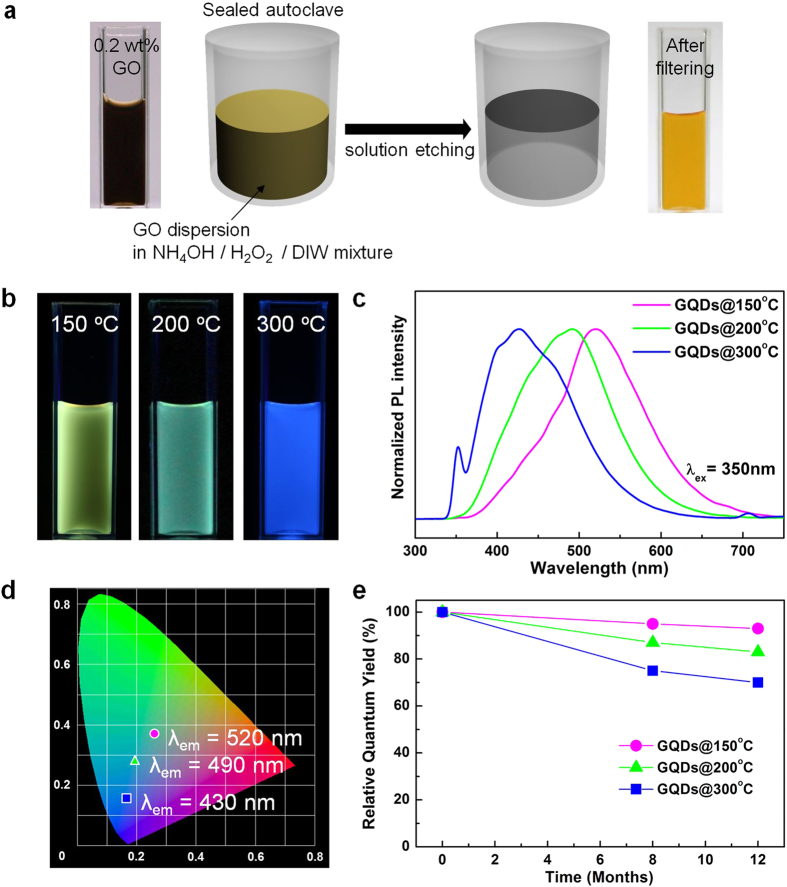
(**a**) Schematic drawing illustrating the solution-phase etching of GO sheets to prepare GQDs. An aqueous GO dispersion (0.2 wt. %) was mixed with NH_4_OH and H_2_O_2_ in a sealed vessel, and was directly heated at 150 °C for 5 h. After allowing the resulting solution to naturally cool, it was filtered. (**b**) Photographs of the GQDs dispersions, prepared at 150, 200, and 300 °C from left to right, under 365 nm UV light. (**c**) Comparison of the PL spectra of the GQDs. (**d**) CIE color coordinates (1931) derived from the emission spectra presented in (**c**). (**e**) Variation of relative quantum yields of GQDs vs. time for 12 months.

Taken together, selective etching of the defective areas and spontaneous extraction of the embedded crystallites are an effective strategy to produce GQDs (Fig. 2a). The observations made during the vapor etching of GO sheets were utilized for developing a solution-based synthesis process for large-scale preparation of GQDs. Figure 2a presents a schematic illustration of the solution phase GQDs synthesis process. A dark brown colored aqueous GO dispersion (0.2 wt%) was mixed with H_2_O_2_ solution and heated at 150 °C in a closed vessel of a Teflon-lined autoclave for 5 h. After the reaction, the solution was clear and exhibited no photoluminescence (PL) response to excitation at 350 nm (Figure S2). As previously confirmed in the vapor etching experiments, H_2_O_2_ is a harsh etchant with peroxide radicals, as a result of which GO was completely decomposed. However, when NH_4_OH was added to the solution mixture, the filtrate was orange under white light (Fig. 2a) and emitted yellow light when exposed to 365 nm UV light (Fig. 2b). As shown in Fig. 2c, under excitation at 350 nm, the emission peak position (λ_em_) of GQDs synthesized at 150 °C (GQDs@150 °C) was at 520 nm. As shown in Raman spectra of Figure S3, GQDs@150^o^C shows a decreased I_D_/I_G_ ratio of 0.80 from 0.86 of GO. It suggests that the etching reaction successfully removes the amorphous regions of GO sheets. Furthermore, changes in the reaction temperature permitted a straightforward control of PL properties. As shown in Fig. 2b, the GQDs synthesized at 200 °C (GQDs@200 °C) and 300 °C (GQDs@300 °C) exhibited green and blue fluorescence, respectively, under UV excitation at 365 nm. The PL spectra (Fig. 2c) obtained by excitation at 350 nm showed a significant red shift of the PL peak position from 430 nm to 520 nm as the reaction temperature was decreased from 300 °C to 150 °C. As drawn in CIE color coordinates (1931) derived from the emission spectra (Fig. 2d), GQDs with diverse emission properties were directly synthesized by controlling the reaction temperature. Indeed, the synthesized GQDs exhibited high QY even without additional modifications, such as polymeric hybridization with polyethylene glycol^19^. As summarized in Table S1, blue, green, and yellow light-emitting GQDs exhibited QYs of 16, 15, and 8.4%, respectively, which are relatively high values compared to previously synthesized graphene-derived QDs^30^,^31^,^35^. More interestingly, the sedimentation of GQD colloids was not observed and the QYs of as-prepared GQDs did not significantly decrease even 12 months after synthesis (Fig. 2e and S4), implying the stable colloidal properties of GQDs.

TEM images of the GQDs synthesized at 150, 200, and 300 °C are shown in Figure S5a–c. A high-resolution TEM (HR-TEM) image (Figure S5d) of a GQD synthesized at 300 °C provides an estimate of its diameter. Further, the image indicates that highly crystalline lattice fringes have a lattice spacing of about 0.21 nm, which is well-matched with the in-plane lattice spacing of (100) planes in graphene (d_100 _= 0.213 nm)^36^. The size distributions (Figure S5e) of the GQDs were plotted by measuring the size of more than 100 GQDs from TEM images. The average diameters of GQDs were calculated to be about 2.0, 2.1, and 2.1 nm for GQDs synthesized at 150, 200, and 300 °C, respectively. Based on these results, it should be noted that the size of the GQDs is not greatly dependent on the synthesis temperature. Therefore, it appears that the large differences in emission behavior of GQDs synthesized at different temperatures are not due to the size of the GQDs, but rather a result of the different chemical functionalities.

**Figure 3:**
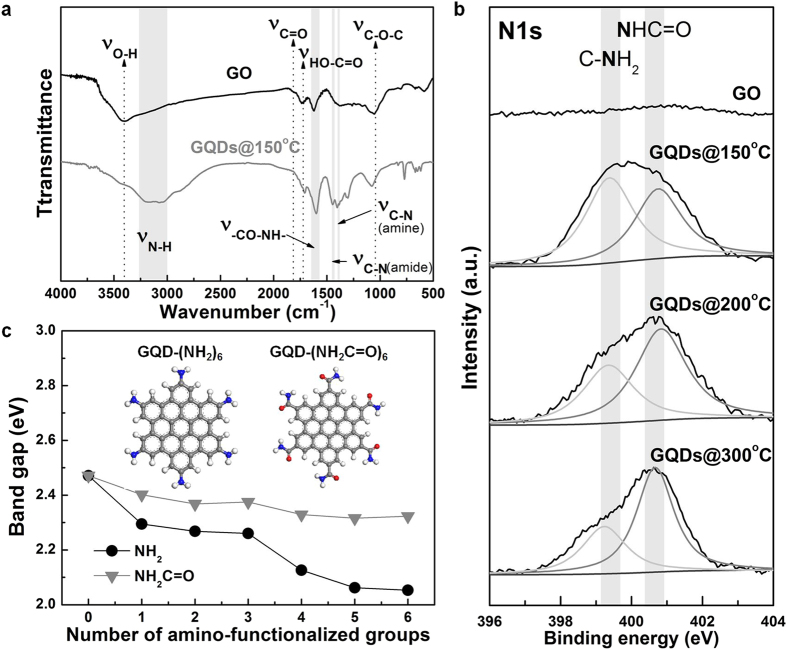
Chemical composition of GQDs. (**a**) FT-IR spectra of GO and GQDs. The IR pattern of GO suggests the presence of oxygen-containing functional groups, such as epoxide, carbonyl, and hydroxyl groups. The IR spectra of GQDs contained new peaks corresponding to amine and amide functionalities. (**b**) XPS N1s spectra of GO and GQDs. Although the XPS spectrum of GO does not show a nitrogen peak, the XPS spectra of GQDs show two new peaks at 399.4 and 400.6 eV, corresponding to primary amine and amide, respectively. (**c**) Band gaps of GQD-(NH_2_)_n_ and GQD-(NH_2_C=O)_n_ as a function of the number of amine and amide functional groups. Gray, white, blue, and red colors on the atomic structures represent carbon, hydrogen, nitrogen, and oxygen, respectively.

The chemical properties of GO and yellow GQDs (synthesis temperature: 150 °C) were compared by analyzing their Fourier-transform infrared (FT-IR) spectra, as shown in Fig. 3a. GO contains various kinds of oxygen-containing functional groups, such as epoxide, carbonyl/carboxyl, and hydroxyl/carboxyl groups exhibiting peaks at 1053, 1820/1735, and 3400 cm^−1^, respectively. Interestingly, in the case of GQDs, the introduction of nitrogen atoms into graphene results in the appearance of peaks corresponding to new vibration modes of C-N stretching (amine/amide, 1400/1450 cm^−1^), C=O stretching (amide, 1600 cm^−1^), and N-H stretching (amine/amide, 3000–3500 cm^−1^). In addition, while GO does not show peaks in the N1s XPS (X-ray photoelectron spectroscopy) spectrum (Fig. 3b), GQDs show peaks at 399.4 and 400.6 eV, which are attributed to primary amine and amide, respectively. From the high-resolution XPS data, N/C atomic ratios of GQDs were calculated (Figure S6). It may be noted that the yellow GQDs (synthesis temperature: 150 °C) were decorated with more amine than amide groups. On the other hand, the major nitrogen functionality of blue GQDs (synthesis temperature: 300 °C) was amide.

Based on these observations, we used quantum mechanical Density Functional Theory (DFT) to understand the underlying fundamental effects of amine and amide functionalities on the optical properties of GQDs. We considered two types of GQD structures functionalized with various numbers ( = 1–6) of amine (GQD-(NH_2_)_n_) or amide groups (GQD-(NH_2_C=O)_n_). The functional groups were located at para (*p*)-sites only because the GQDs with amine and amide groups at the para (*p*)-sites exhibited lower energy compared to the corresponding GQDs with amine and amide groups at meta (*m*)-sites (Figure S7). Among various configurations (Figures S8 and S9), we focused on the structure with the lowest energy for each number of functional groups and calculated the band gaps of GQDs after geometry optimization, as shown in Fig. 3c. The band gaps of both the GQDs tend to gradually decrease with an increase in the number of functional groups. The band gap of GQD-(NH_2_)_6_ (2.053 eV) was significantly decreased compared to that of pure GQD (2.471 eV), indicating red shift. Meanwhile, GQD-(NH_2_C=O)_6_ (2.323 eV) showed a band gap shift of −0.148 eV from that of pure GQD. The results indicate that the presence of amine functional groups on the GQDs leads to a stronger tendency for shifting λ_em_ towards longer wavelengths than that of amide functional groups. Therefore, GQDs containing more amine functionalities emit yellow light, whereas GQDs prepared at higher temperatures emit blue light, owing to fewer amine functionalities in the latter case. Therefore, we conclude that the difference in optical properties of the GQDs prepared at different temperatures originates from the chemical functionalities.

**Figure 4:**
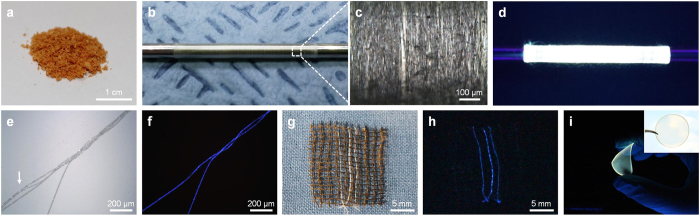
(**a**) Photograph of freeze-dried GQD powders. Images of long GQD fibers taken up on a steel drum (diameter, 7 mm) under (**b**) low magnification and (**c**) high magnification. (**d**) GQDs fibers emitting a strong yellow light under UV lamp (365 nm). Optical microscopic images of blue GQD/PAA composite fibers entwisted with PAA fibers under (**e**) bright-field conditions and (**f**) UV illumination. Photographs of three strands of GQD yarn woven with a commercial cotton fabric under (**g**) white light and (**h**) UV irradiation. The white arrow in (**e**) points to the PAA fibers. (**i**) Photographs of a yellow GQD/PAA composite film under UV lamp (365 nm) and (inset) white light.

Table 1 compares GQDs fabricated in this work with those reported in other literatures. Yellow GQDs (synthesized at 150 °C), green GQDs (synthesized at 200 °C), and blue GQDs (synthesized at 300 °C) were obtained with synthesis yields of about 70 wt%, 72 wt%, and 58 wt%, respectively. Our strategy provides a straightforward route for synthesizing GQDs in large quantities and could be beneficial for potential applications in the synthesis of GQD-based composites with unique emitting properties. Figure 4a shows a photograph of freeze-dried GQD powders (about 50 mg) prepared from 40 mL of GO solution (0.2 wt%). To demonstrate the potential applications of GQDs in composites, GQD powders were mixed with poly(acrylic acid) (PAA) and the viscous GQDs-PAA mixture was hand-drawn to prepare microfibers. To date, the construction of QD-based polymeric composites is a huge challenge because the redispersion of solidified QDs was largely limited due to its severe aggregation and the composites often showed low optical activities. Our GQD powders were successfully dispersed in a polymeric matrix at a high concentration and the several meters long mechanically flexible GQDs fibers were readily taken up on a steel rod (Fig. 4b). On the surface of steel drum, fibers with about 5 μm in diameter containing GQDs@150 °C are clearly shown (Fig. 4c). Under 365 nm UV excitation, GQD fibers are emitting the strong yellow light as shown in Fig. 4d. For comparison, PAA fibers were also prepared in the same manner without adding GQD powders and twisted with GQD fibers (indicated by the white arrow in Fig. 4e). Under UV excitation, fibers containing GQDs synthesized at 300 °C emitted strong blue light, whereas non-fluorescent PAA fibers were not detectable (Fig. 4f). In addition, mechanically stable GQD fibers were interwoven in other textiles and solely emitted blue light (Fig. 4g,h). GQDs nanocomposites were also drop-casted in a circular-shaped mold having a diameter of 3.5 cm. Figure 4i shows the yellow light emitting GQDs-PAA composite films containing GQDs synthesized at 150 °C. As shown in the inset, the film was almost transparent under white light, whereas it exhibited fluorescence upon UV irradiation. It may be noted that the emitted light from the films was not localized, indicating that GQD powder was homogeneously mixed within the PAA matrix.

## Conclusions

In this study, we have showed that GO is a promising platform for the preparation of GQDs. GO has a unique molecular structure with a mixture of crystalline graphitic region and amorphous defect sites. The extraction of graphite domains from GO could be an effective pathway for the preparation of GQDs. Motivated by the successful gas-phase etching on GO, a solution-based hydrothermal reaction was designed by introducing H_2_O_2_ and NH_3_. GQDs with high QYs and high process yields were synthesized through the designed processes, which did not involve any complicated procedures. Our straightforward route provides a highly efficient method for producing optically tuned GQDs for applications in polymeric composites such as fibers or films.

## Materials and Methods

### Synthesis of GO

GO was prepared from graphite powder (Bay Carbon, SP-1) using the modified Hummers method, which has been reported elsewhere^37^,^38^,^39^. GO was washed with 1 M HCl by vacuum-assisted filtration and was then dialyzed using a dialysis membrane (Spectra Dialysis Membrane, MWCO: 6,000–8,000) for 15 days to remove the salt byproduct and excess acid. GO powders were obtained by freeze-drying the aqueous GO solution using a lyophilizer (Ilshin, FD 8518).

### Vapor-phase etching of GO

The prepared GO was deposited on SiO_2_/Si substrates using the Langmuir-Blodgett technique^6^,^32^. GO-deposited SiO_2_/Si substrates were suspended in a glass pipette and placed in a 50 mL Teflon vessel pre-filled with a solution mixture containing 10 mL of deionized water (DIW; Millipore, Direct Q3), 0.5 mL of NH_4_OH (Sigma-Aldrich, 30% NH_3_ basis), and 1 mL of H_2_O_2_ (Sigma-Aldrich, 30% (w/w) in H_2_O). The Teflon vessel was sealed in an autoclave and heated to 150 °C. To observe the intermediate stages of steam etching, GO sheets suspended on a lacey carbon grid were exposed to water vapor at 200 °C for 20 h.

### Solution-phase etching of GO

The GO powder (0.2 wt%) was re-dispersed in 10 mL of DIW by mechanical agitation. Subsequently, 0.5 mL of NH_4_OH and 1 mL of H_2_O_2_ were added to the dispersion and the mixture was further stirred for 10 min at room temperature. The mixture was then transferred to a 50 mL Teflon vessel and sealed in an autoclave. After heating at 150 °C – 300 ^o^C for 5 h, the solution was naturally cooled down to room temperature and filtered with a 0.02-μm syringe filter (Whatman, Anotop 10) to remove any flocculated aggregates. The filtrate was stirred and heated at 100 °C for 1 h to remove any remaining NH_3_.

### Preparation of GQD fibers and films

10 mg of freeze-dried GQD powders and 1 g of poly(acrylic acid) (PAA; Sigma-Aldrich, M_v_  ~ 450,000) were mechanically mixed in 6 mL of DIW, and the viscous mixture was degassed at room temperature under vacuum for 1 h to remove bubbles. GQD fibers were spun from the viscous mixture. Bare PAA fibers were also fabricated by following the same method without adding GQD powders. GQD films were prepared by drop-casting the mixture on a circular-shaped mold having a diameter of 3.5 cm. The spun fibers and films were dried at ambient conditions to remove any residual water.

### Characterization

GO sheets and GQDs were characterized using an atomic force microscopy (AFM; Park Systems, XE-70) in the tapping mode and a transmission electron microscopy (TEM; JEOL, JEM-2100F) operated at an accelerating voltage of 200 kV. TEM samples were prepared on lacey-carbon TEM grids (Ted Pella, Inc.). PL and QY measurements were carried out using a fluorescence spectrometer (Scinco, FS-2) and a UV-vis spectrometer (Analytik Jena, Specord200). In order to measure long-term stability, as-prepared GQDs were kept in solution state under ambient conditions over 12 months. Photographs of fluorescent GQD solutions and composite films and fibers were taken using a digital camera (Canon, EOS 600D) with a 356 nm UV lamp (Specroline, ENF-260C/FE). The fluorescent GQD fibers were observed using a fluorescence microscopy (Olympus, BX51/U-RFL-T) with a UV excitation filter (Olympus, U-MNU2). X-ray photoelectron spectroscopy (XPS) measurements were carried out using a spectrometer (Thermo Scientific, Theta probe) with monochromatic Al Kα radiation. Fourier-transform infrared spectroscopy (FT-IR; Thermo Scientific, Nicolet 6700) was conducted with KBr pellets. Raman spectroscopy (JASCO, NRS-3100) was performed with 514 nm laser excitation.

### Computational Details

Fully optimized structures of GQDs were obtained using DFT calculations. All the first-principle calculations were carried out using the DMol^3^ package^40^,^41^ of Material Studios from Accerlys^42^ with generalized gradient approximation (GGA). The Perdew-Burke-Ernzerhof (PBE) functional^43^,^44^ was used to treat the electron exchange-correlation energy of interaction of the electrons, employing the double numerical basis with polarization *d* and polarization *p* functions on hydrogen (DNP). Self-consistent field (SCF) convergence of 10^−5^ Ha was obtained with convergence tolerances of 0.002 Ha/Å for the total force and 0.005 Å for the maximum displacement. Spin polarized calculations were performed in this study. The real space cutoff radius was set to 3.7 Å. The band gap for each configuration of amino-functionalized GQDs refers to the energy difference between the highest occupied molecular orbitals (HOMO) and the lowest unoccupied molecular orbitals (LUMO).

## Additional Information

**How to cite this article**: Park, H. *et al.* Large Scale Synthesis and Light Emitting Fibers of Tailor-Made Graphene Quantum Dots. *Sci. Rep.* **5**, 14163; doi: 10.1038/srep14163 (2015).

## Supplementary Material

Supplementary Information

## Figures and Tables

**Table 1:** Comparison of GQDs synthesized from various methods.

**Precursor**	**Synthetic method**	**Quantum yield (%) (Color)**	**Production yield (wt%)**	**Ref.**
Graphene oxide	Selective elimination of defective (amorphous) area (Top-down)	8 (Yellow)	70	this work
		15 (Green)	72	
		16 (Blue)	58	
Graphite	Oxidative cutting/post-functionalization (Top-down)	2 (White)	N/A	45
Graphite	Intercalation compound-assisted exfoliation (Top-down)	4 (Blue)	N/A	46
Carbon soot	Thermal plasma jet (Top-down)	13 (Blue)	N/A	47
Graphite	Electrochemical oxidation (Top-down)	N/A	40	48
Glucose	Microwave-assisted hydrothermal (Bottom-up)	11 (Blue)	78	49
Glycerol	Microwave-assisted pyrolysis (Bottom-up)	N/A	10	50
