# Supplementary material for: Large Scale Synthesis and Light Emitting Fibers of Tailor-Made Graphene Quantum Dots
Source: Sci Rep. 2015 Sep 18;5:14163. doi: 10.1038/srep14163 (PMC4585659; doi:10.1038/srep14163)
Supplement: Supplementary Information [file srep14163-s1.pdf]

Supplementary Information

Large Scale Synthesis and Light Emitting Fibers of Tailor-Made Graphene Quantum Dots

Hun Park[1], Sung Hyun Noh[1], Ji Hye Lee[2], Won Jun Lee[3], Jae Yun Jaung[1], Seung Geol Lee[2,*] and, Tae Hee Han[1,*]

[1]*Department of Organic and Nano Engineering, Hanyang University, Seoul 133-791, Republic of Korea*

[2]*Department of Organic Material Science and Engineering, Pusan National University, Busan 609-735, Republic of Korea*

[3]*Department of Chemistry, Imperial College London, London, SW7 2AZ, United Kindgom*

[*]Corressponding author: seunggeol.lee@pusan.ac.kr (S.G. Lee), than@hanyang.ac.kr (T.H. Han)

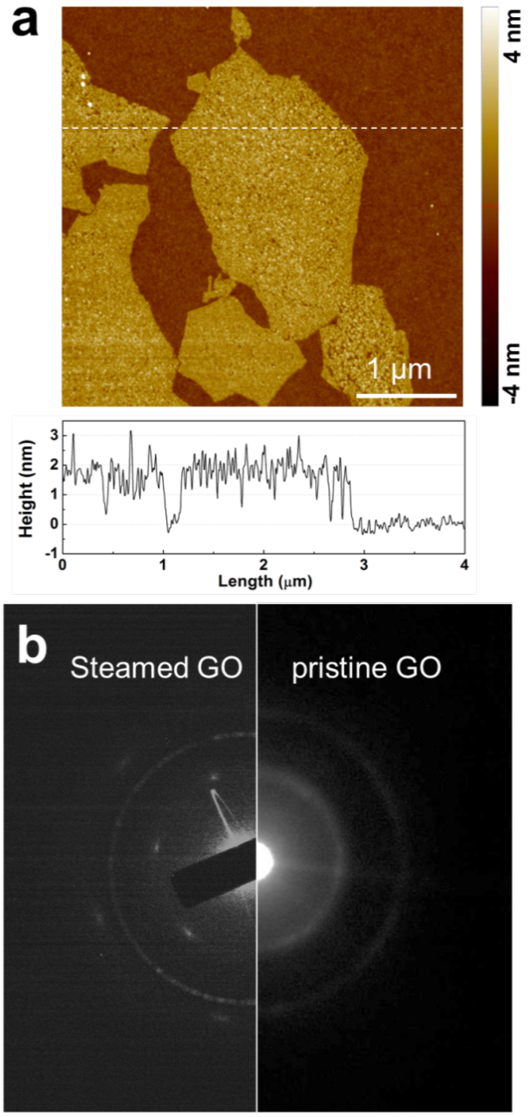

Figure S1. (a) AFM image of GO sheets etched with a H_2O_2/DIW solution mixture for 25 min. The corresponding height profiles are measured along the white lines. (b) Electron diffraction patterns of the steam etched GO sheets (left) and a pristine GO sheet (right).

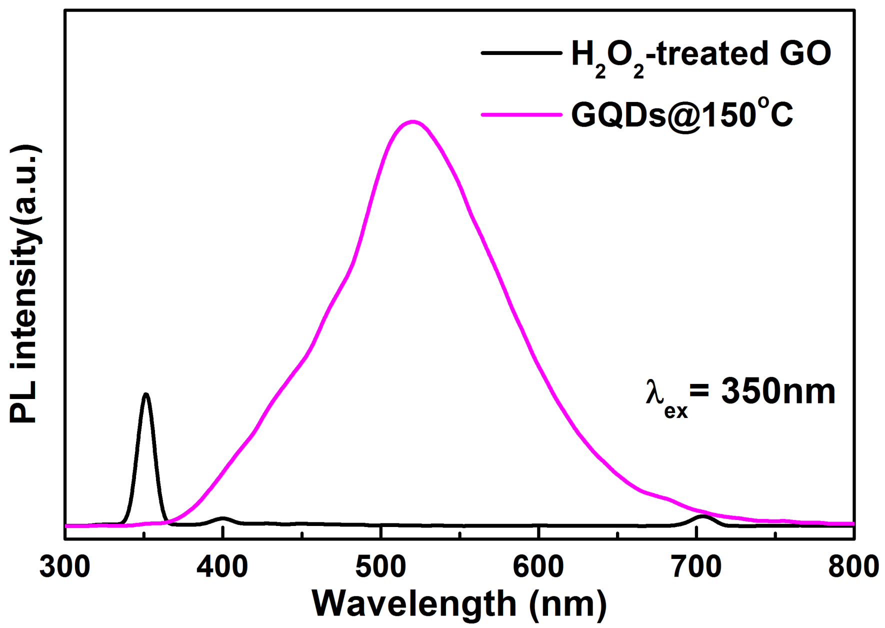

Figure S2. PL spectrum of H_2O_2-treated GO at 150°C for 5 hours. It does not show any PL response under 350 nm excitation. For comparison of the PL intensity, PL spectrum of GQDs@150°C is also presented.

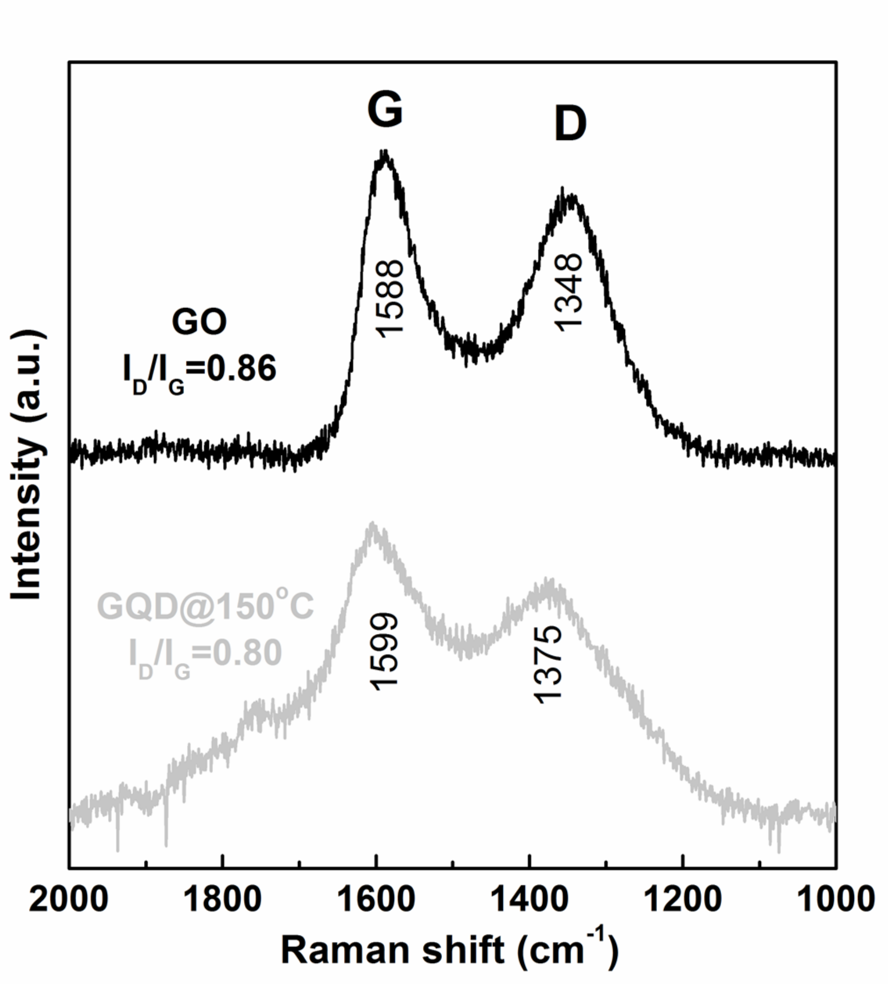

Figure S3. Raman spectra of GO and GQD@150°C.

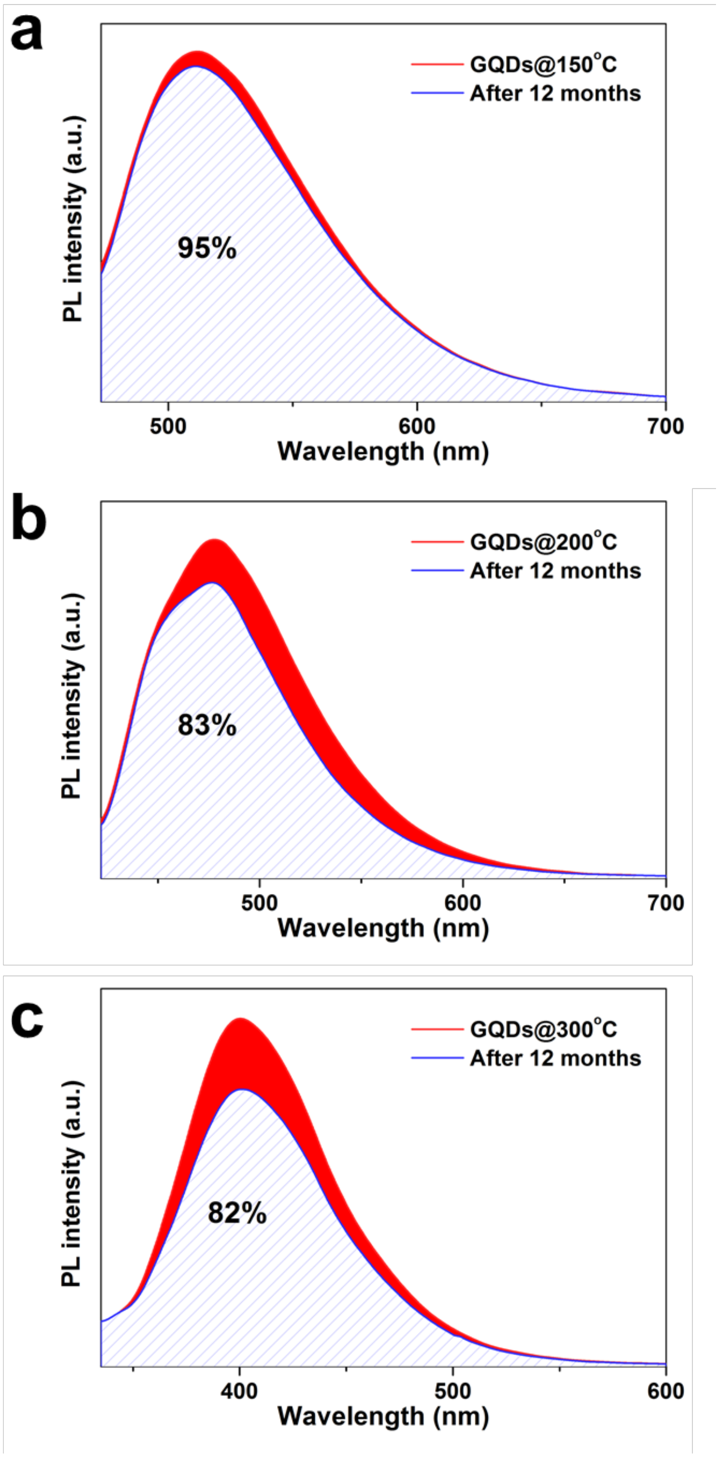

Figure S4. Long-term stability of GQDs. Comparison of PL spectra between fresh GQDs and the same GQDs stored for 12 months in an ambient condition (a, b, c). The area ratio of the 12-months-stored GQDs to fresh GQDs is presented.

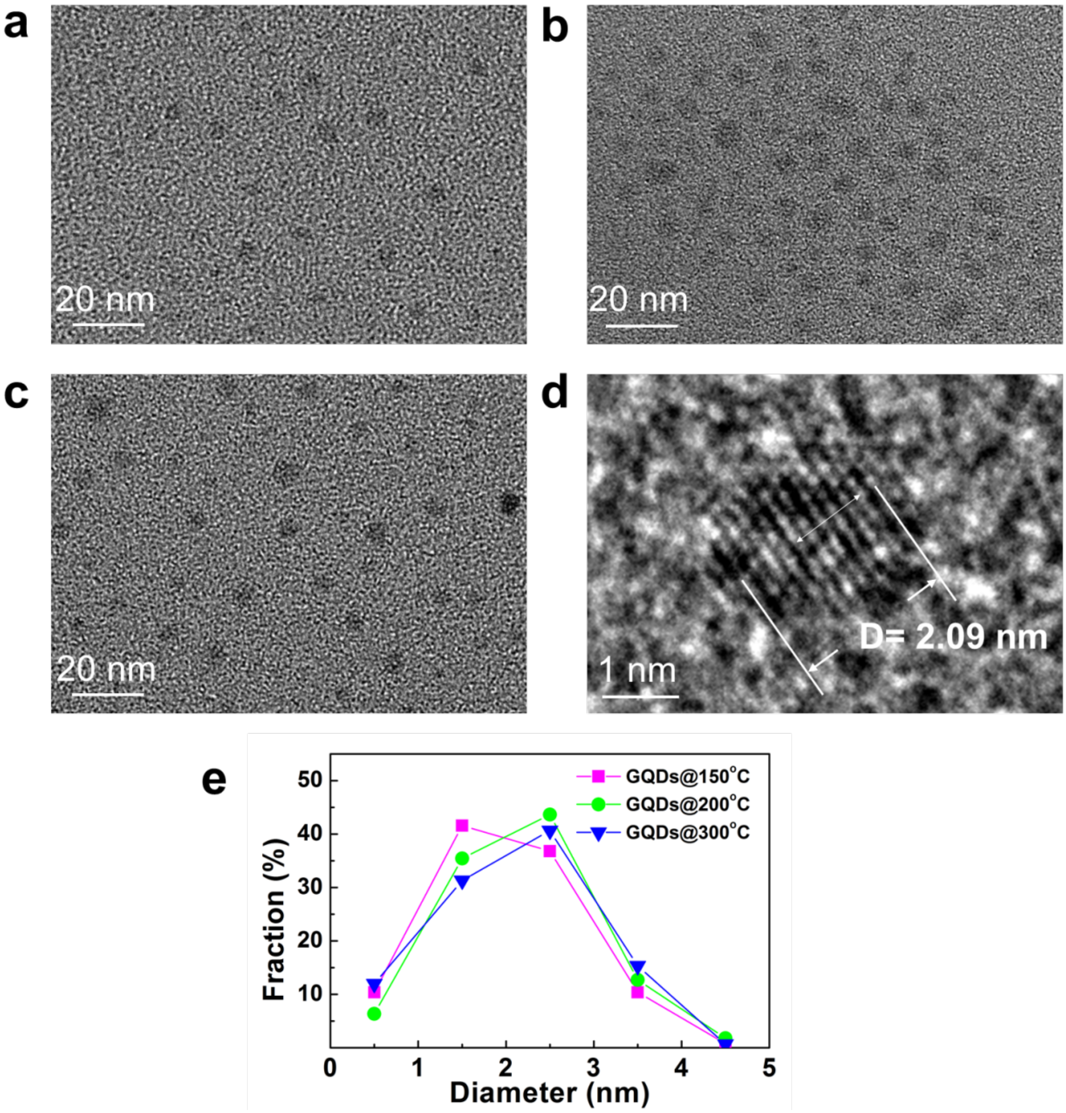

Figure S5. TEM images of GQDs prepared at (a) 150 °C, (b) 200 °C, and (c) 300 °C. (d) HR-TEM image of a GQD synthesized at 300 °C. (e) Size distributions of GQDs.

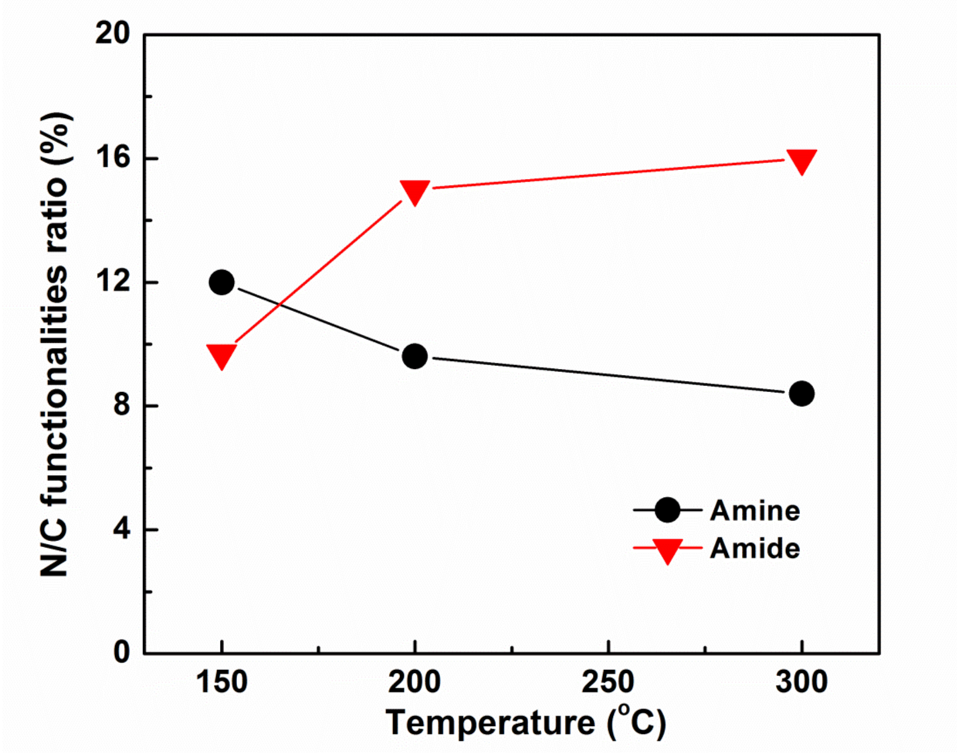

Figure S6. Amine and amide functionalities ratio of GQDs vs. reaction temperature, derived from XPS analysis.

Table S1. Quantum yield (QY) of GQDs. Quinine sulfate (QY= 0.54) in 0.5 M H_2SO_4 was chosen as the reference standard for the blue-fluorescent GQDs, coumarin 153 (QY= 0.38) in ethanol for the yellow one, and perylene (QY= 0.92) in ethanol for the green one. The quantum yield of GQDs in water was calculated from UV-vis absorption and PL spectra according to the following equation:

$$\Phi_x = \left(\frac{Ax}{Ar}\right)\left(\frac{Fx}{Fr}\right)\left(\frac{nx}{nr}\right)^2 \Phi_r$$

Where Φ, A, F, and n are the quantum yield, the absorbance at the excitation wavelength, the area under the emission curve, and the refractive index of the solvents used, respectively. Subscript r and x refer to the reference standard and to GQDs, respectively.

	A	F	n	Φ
GQDs@150°C	0.43	3.83412x 10^4	1.33 (H_2O)	8.4 %
GQDs@200°C	0.46	7.44760x 10^4	1.33 (H_2O)	15 %
GQDs@300°C	0.41	8.82182x 10^4	1.33 (H_2O)	16 %

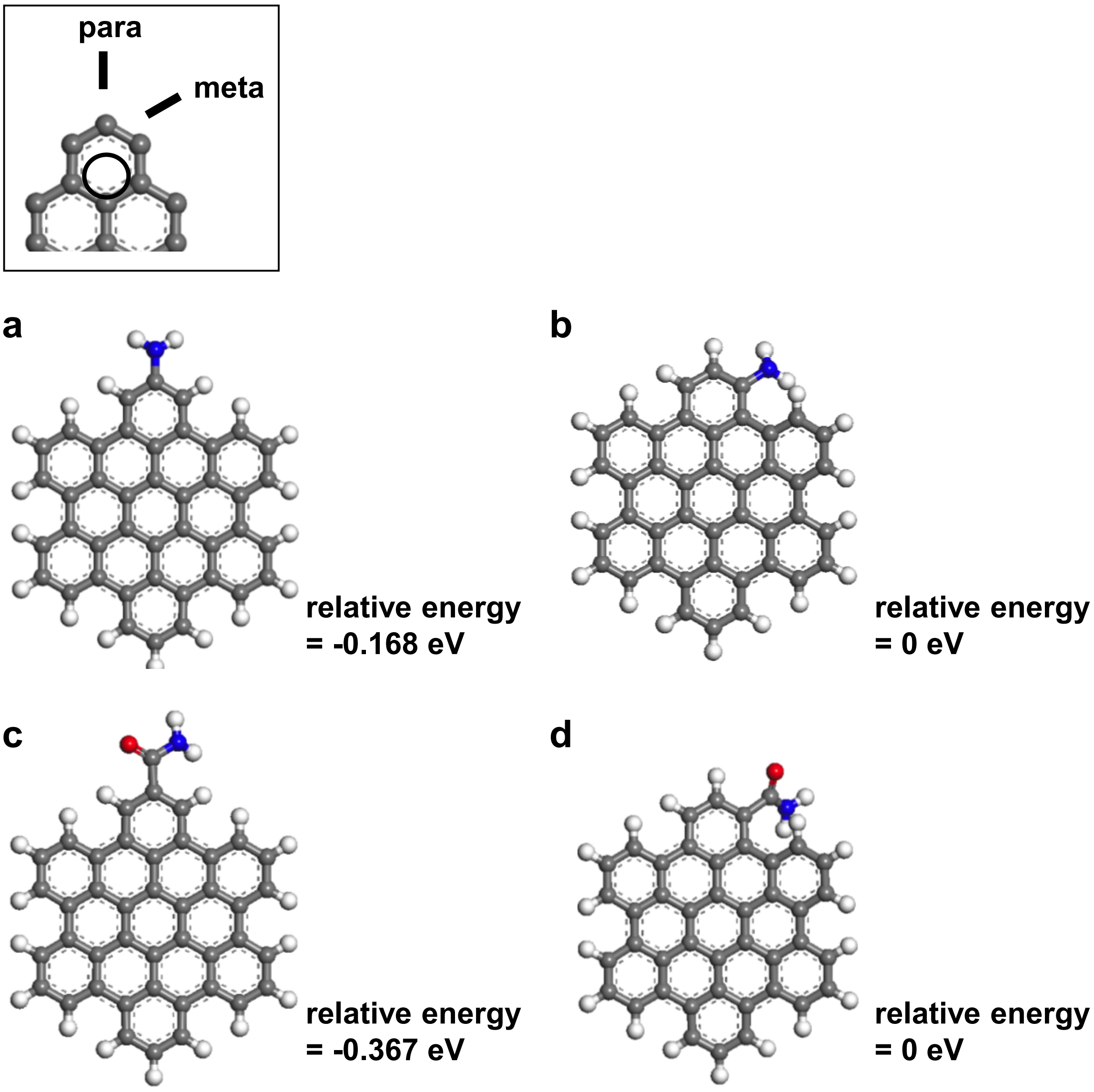

Figure S7. The optimized structures of GQD-NH_2 and GQD-NH_2C=O with two different edge types, para(*p*)-site and meta(*m*)-site: GQD-NH_2 with (a) para(*p*)-site and (b) meta(*m*)-site; GQD-NH_2C=O with (c) para(*p*)-site and (d) meta(*m*)-site.

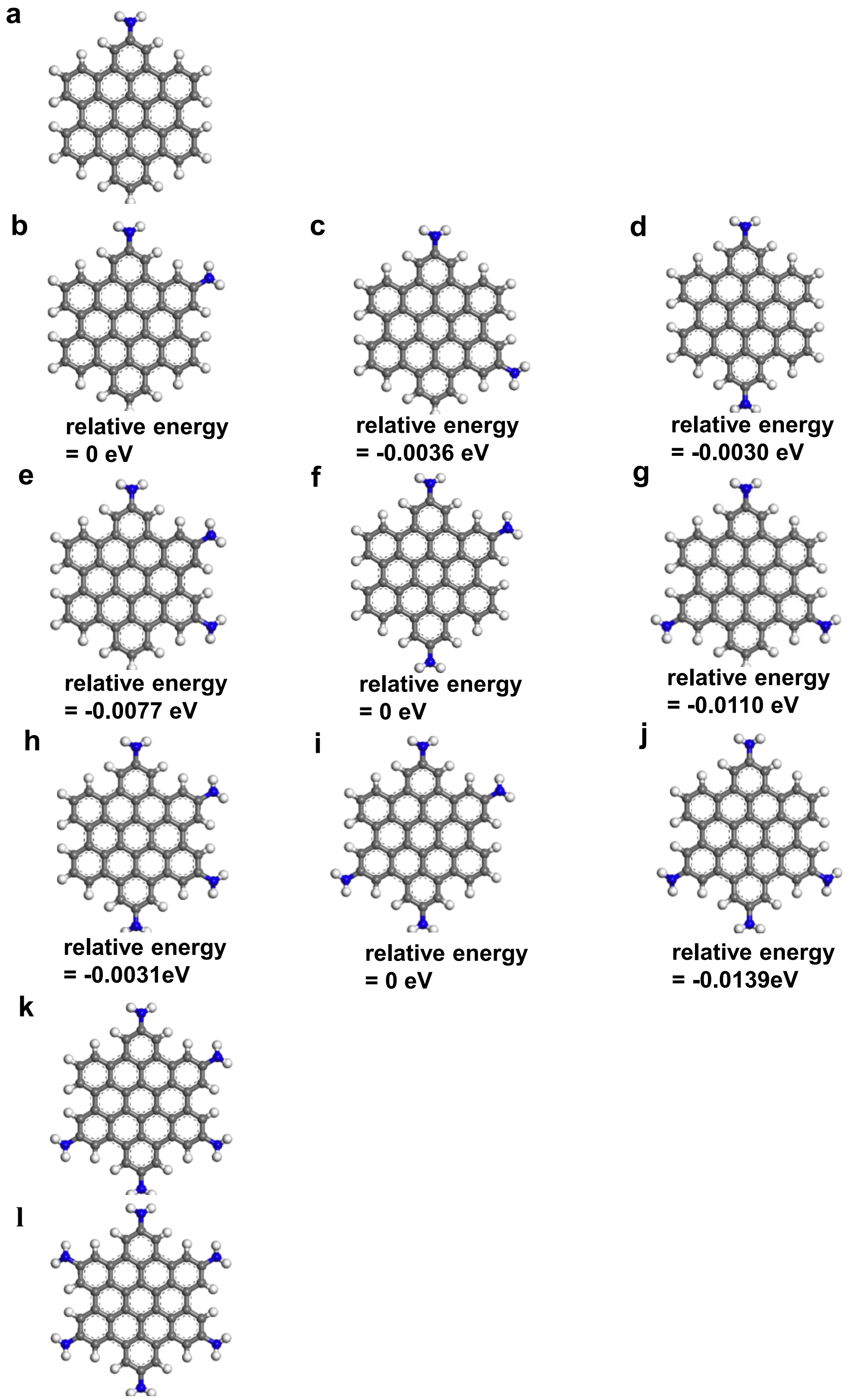
a
b
relative energy
= 0 eV
c
relative energy
= -0.0036 eV
d
relative energy
= -0.0030 eV
e
relative energy
= -0.0077 eV
f
relative energy
= 0 eV
g
relative energy
= -0.0110 eV
h
relative energy
= -0.0031eV
i
relative energy
= 0 eV
j
relative energy
= -0.0139eV
k
l

Figure S8. All of the possible configurations of GQD-$(NH_2)_n$ with para(*p*)-site as a function of the number of the amine functional group: (a) GQD-$(NH_2)_1$; (b)-(d) GQD-$(NH_2)_2$; (e)-(g) GQD-$(NH_2)_3$; (h)-(j) GQD-$(NH_2)_4$; (k) GQD-$(NH_2)_5$ and (l) GQD-$(NH_2)_6$.

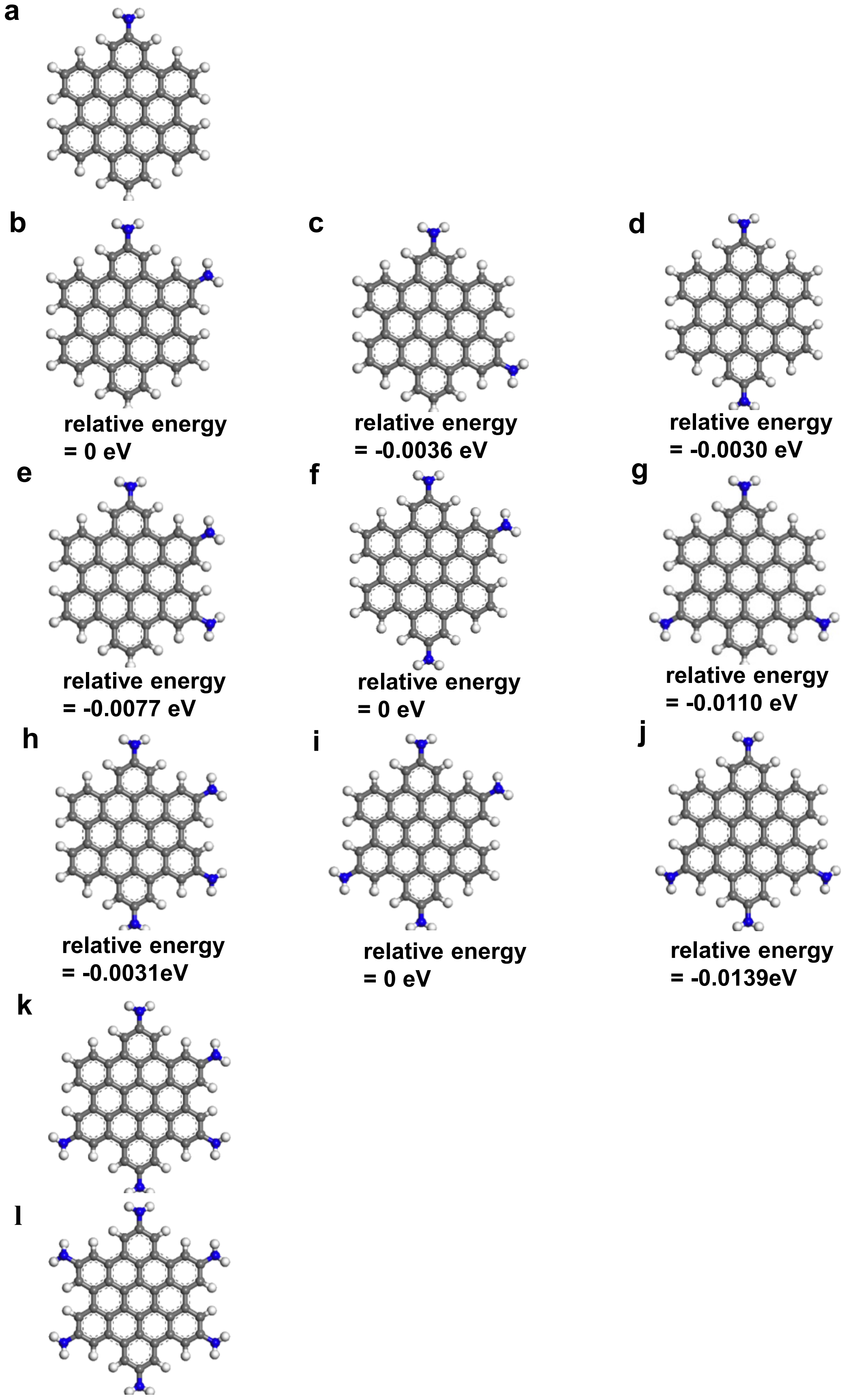

a
b
relative energy
= 0 eV
c
relative energy
= -0.0036 eV
d
relative energy
= -0.0030 eV
e
relative energy
= -0.0077 eV
f
relative energy
= 0 eV
g
relative energy
= -0.0110 eV
h
relative energy
= -0.0031eV
i
relative energy
= 0 eV
j
relative energy
= -0.0139eV
k
l

Figure S9. All of the possible configurations of GQD-($NH_2C=O$)$_n$ with para(*p*)-site as a function of the number of the amide functional groups: (a) GQD-($NH_2C=O$)$_1$; (b)-(d) GQD-($NH_2C=O$)$_2$; (e)-(g) GQD-($NH_2C=O$)$_3$; (h)-(j) GQD-($NH_2C=O$)$_4$; (k) GQD-($NH_2C=O$)$_5$ and (l) GQD-($NH_2C=O$)$_6$.
